# Learning best practices for educating a caregiving workforce

**DOI:** 10.1093/geroni/igab046.3096

**Published:** 2021-12-17

**Authors:** Sweta Tewary, Yumna Indorewala, Nicole Cook, Naushira Pandya, Sashah Damier, Assma Twahir

**Affiliations:** 1 Nova Southeastern University, Fort Lauderdale, Florida, United States; 2 NSU, NSU, Florida, United States; 3 NSU, Davie, Florida, United States; 4 NSU, Davie,FL, Florida, United States

## Abstract

It is well established that the health professional workforce is not adequately prepared to meet the demands of an aging older population. Caregivers are often the backbone supplemental workforce for seniors, providing daily care with assistance with activities of daily living, with little training. Part of the mission of the South Florida Geriatric Workforce Enhancement Program (SFGWEP) is to support and empower caregivers through community based training programs. Between January 1 2020 to January 31, 2021 SFGWEP provided education to more than 340 caregivers on topics related to opioid use, effective communication with individuals with dementia and other topics. Attendees responded to a short evaluation survey, which included three multiple-choice questions on if attending was a good use of their time, if they gained knowledge and if they plan to apply material, and two open-ended questions to identify opportunities for improvement in future trainings. Responses were overwhelmingly positive (>98% for multiple-choice questions.) There were also three open-ended questions that were analyzed using a modified thematic approach. The three questions covered what attendees learned, what they wanted to learn more about and suggestions for improvement. Analysis suggests that attendees plan to be more mindful about communication (e.g. improve eye contact, listen more) and that they want more information on neurocognitive disorders and resent research, including psychological changes due to disease and medication side effects. In terms of improvement, attendees said the program should allow more time for questions and should use more engaging materials (polls, posters, flyers and case studies).

